# Detection and Molecular Characterization of a Natural Coinfection of Marek’s Disease Virus and Reticuloendotheliosis Virus in Brazilian Backyard Chicken Flock

**DOI:** 10.3390/vetsci6040092

**Published:** 2019-11-20

**Authors:** Ruy D. Chacón, Claudete S. Astolfi-Ferreira, Marta B. Guimarães, Luciana N. Torres, David I. De la Torre, Lilian R. M. de Sá, Antonio J. Piantino Ferreira

**Affiliations:** Department of Pathology, School of Veterinary Medicine, University of São Paulo, Av. Prof. Orlando M. Paiva, 87, CEP 05508-270 São Paulo, Brazil; ruychaconv@usp.br (R.D.C.); csastolfi@gmail.com (C.S.A.-F.); mbrito@usp.br (M.B.G.); lu.patologia.fmvz@gmail.com (L.N.T.); daviddelatorreduque@gmail.com (D.I.D.l.T.); liliansa@usp.br (L.R.M.d.S.)

**Keywords:** Marek’s disease virus (MDV), reticuloendotheliosis virus (REV), molecular characterization, sequencing, phylogenetic analysis

## Abstract

Marek’s disease virus (MDV) and the reticuloendotheliosis virus (REV) are two of the primary oncogenic viruses that significantly affect chickens. In Brazil, there have been no previous published reports on the presence of field REV alone or in coinfection. This retrospective study analyzes samples from a case of lymphoproliferative lesions from a backyard chicken flock. MDV and REV were detected by PCR and classified as MDV1 and REV3, respectively, through sequencing and phylogenetic analysis based on the glycoprotein B (gB) genes for MDV and the polymerase (*pol*) and envelope (*env*) genes for REV. Real-time PCR reactions were performed for MDV to rule out the presence of the Rispens vaccine strain. This is the first report of the presence of REV in coinfection with a MDV clinical case in Brazil and the first molecular characterization of REV in South America. This study highlights the importance of molecular diagnosis for REV and MDV in poultry. In addition, this study highlights the distribution of these two viruses worldwide and the latent risk of them solely or in coinfection to this part of the world.

## 1. Introduction

In chickens, neoplastic diseases typically have a viral etiology and cause a significant economic impact [1,2,3]. The three primary disease-causing agents are the Marek’s disease virus (MDV), the avian leukosis virus (ALV), and the reticuloendotheliosis virus (REV). The prototype MDV is the Gallid alphaherpesvirus 2 (serotype 1, MDV1) and is grouped in the Mardivirus genus together with the following relative strains: The Gallid alphaherpesvirus 3 (serotype 2, MDV2) and Meleagrid alphaherpesvirus (serotype 3, MDV3), according to the latest information released on the Herpesviridae family at the International Committee on Taxonomy of Viruses (ICTV) [4].

Infections caused by MDV1 are associated with lymphoproliferative lesions, which can include nervous affection, paralysis, bursal atrophy, parenchymal neoplastic cellular infiltrations, pleomorphic lymphomas, and neoplastic cells infiltration in several visceral organs, nerves, muscles, and the skin [5]. Pathotypic classification for MDV designates the following 4 groups: mild (mMDV), virulent (vMDV), very virulent (vvMDV), and very virulent plus (vv+MDV) [1].

ALV is an Alpharetrovirus, and it is the most representative species of this genus. Strain classification of ALV that infects chickens includes six subgroups (A–J), which are classified according to the antigenicity of the viral envelope glycoproteins; depending on the group, the virus can cause lymphoid or myeloid leukosis. Tumors are usually nodular, and infiltrations are composed of uniform neoplastic infiltrations of lymphoblasts [2].

REV is a Gammaretrovirus that is associated with runting-stunting syndrome, neoplasia of lymphoid and other tissues, acute reticulum cell neoplasia, and immunodepression [3]. All known reported REV strains are strongly related serologically, but they are divided into subtypes 1 (REV1, prototype REV-T), 2 (REV2, prototype Spleen Necrosis Virus (SNV)), and 3 (REV3, prototype Chicken Syncytial Virus (CSV)) [6].

The classical methods for the differential diagnosis of lymphoid neoplastic diseases in birds are viral isolation and histopathological examinations; however, due to the technical difficulties of the virus isolation and the absence of pathognomonic microscopic lesions, molecular or immunohistochemical tests are often required [5]. Usually, diagnostic and research laboratories process and may store tissues for several years. Because of this storage practice, techniques have been developed to perform PCR for oncogenic viruses [7,8].

Coinfection and the integration of the partial or total genome of REV into other avian viruses, such as MDV [9,10] and fowlpox (FPV) [11,12] virus, were discovered several years ago. These mutagenesis events can alter the biological functions of the viruses involved, including field strains or even vaccine strains [13,14,15]. The presence of MDV worldwide, primarily in countries with a poultry industry, is detected and generally controlled with the use of commercial vaccines [1]. On the other hand, reports of REV infections are much less common and ubiquitous in field samples.

Brazilian backyard flocks are usually not vaccinated against most of the avian viruses and even isolated cases of disease are not properly communicated. In South America, antibodies against REV have been detected alone in Peru by ELISA test [16] or in coinfection with MDV in Argentina by Agar gel precipitation (AGP) tests and fluorescent antibodies assays [17]. However, these reports did not provide molecular characterization of these viruses.

The present study reports for the first time in Brazil the presence of REV in coinfection with MDV in a clinical case. In addition, this study presents the first molecular characterization of REV in South America.

## 2. Materials and Methods

### 2.1. Clinical History, Gross Lesions, and Histopathologic Examination

**Figure 1:**
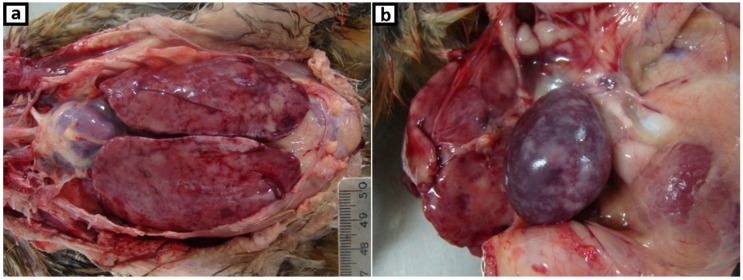
Gross, hen, 12-weeks-old. Multifocal to coalescing white foci associated with (**a**) marked enlarged liver, (**b**) marked enlarged spleen.

A retrospective study was designed to clarify a case (USP386) that corresponds to samples of a twelve-week-old domestic hen from a backyard chicken flock located in São Paulo State in 2010. This flock consisted of 40 birds not vaccinated against MDV and the clinical symptoms observed in the farm were apathy, loss of appetite, and facial cyanosis. Four birds died 3 days after detecting the symptoms and were discarded by the farmer. Approximately half of the flock began to show the same symptoms, and the hen referred was taken to the School of Veterinary Medicine (USP) for necropsy. Gross lesions examination highlighted regular body condition (weight of bird—1.08 kg), marked splenomegaly and hepatomegaly with multifocal to coalescing white foci and diffuse white areas (Figure 1a,b); dark lungs; diffuse thickening of proventricular and small intestine mucosa wall; bilateral increased thickness of peripheral nerves. Samples of liver, spleen, kidney, lung, trachea, proventriculus, gizzard, small intestine, peripheral nerves, and ganglion were fixed in 10% formalin, embedded in paraffin, cut into 5-µm sections, and stained with hematoxylin and eosin (H&E) for light microscopic examination. Otherwise, samples of liver, spleen, proventriculus, and small intestine were frozen at −80 °C since 2010.

### 2.2. Detection of MDV and REV Viruses through PCR Examination

Commercial vaccines (CVI988/Rispens, SB-1, and HVT) were used as positive controls for the MDV serotypes and synthetic DNA fragments (Invitrogen™ GeneArt™ Strings™) in the case of REV and ALV. Spleen from specific-pathogen-free (SPF) chicken resuspended in 1.5 mL of phosphate-buffered saline (PBS) was used as a negative control. DNA extraction of the controls and samples of liver, spleen, proventriculus, and small intestine were performed using the DNeasy Blood & Tissue Kit (Qiagen, Hilden, Germany) according to the manufacturer’s specifications. The eluted suspensions were quantified with a NanoDrop One (ThermoFisher Scientific, Carlsbad, CA, USA) and stored at −80 °C for the subsequent PCR procedures. PCR methods for the detection of MDV and REV were performed as described previously (Table 1) [8]. An RT-Nested-PCR reaction for the detection of ALV was performed as previously described (Table 1) [18].

### 2.3. Real-Time PCR to Detect and Differentiate CVI988 from the Field MDV Serotype

To rule out the presence of the MDV1 type vaccine (CVI988), two SYBR Green based real-time PCR tests (Fast SYBR™ Green Master Mix, Applied biosystems, Austin, TX, USA) were performed on all samples, which tested positive for MDV detection in Section 2.2 with thermal conditions according to a previously described method, which targets a single nucleotide polymorphism (SNP) of nucleotide 320 in the pp38 gene [19]. The non-CVI988 reaction uses the Non-CVI988-F and the MDV pp38-R primers (Table 1) and is specific for amplification of MDV1 strains different of CVI988. The CVI988 specific reaction uses the CVI988-F and the MDV pp38-R primers (Table 1) and is specific for amplification of the Rispens vaccine.

### 2.4. PCR Amplification of the Glycoprotein B Gene of the MDV Serotypes

To confirm the MDV serotype by sequencing, a PCR method was developed using generic primers (Table 1) to amplify a fragment of glycoprotein B (gB) that is common to all three MDV serotypes (MDV-1: Gallid herpesvirus 2, MDV-2: Gallid herpesvirus 3, MDV-3: Meleagrid herpesvirus 1), using the reference sequences (NC_002229, NC_002577, and NC_002641, respectively). The volume of the reaction mix was 25 µL, including 50 ng of template DNA, 0.2 mM of each dNTP, 2 mM MgCl_2_, 0.5 µM of each primer, 1× PCR buffer, and 1 U of Platinum *Taq* DNA Polymerase (ThermoFisher Scientific, Carlsbad, CA, USA). The thermal conditions included an initial denaturation step at 94 °C for 3 min, followed by 35 cycles of 94 °C for 60 s, 54 °C for 60 s, and 72 °C for 90 s, followed by a final elongation step at 72 °C for 10 min.

### 2.5. PCR for the Gag, Polymerase, and Envelope Genes of REV

To confirm the integrity of the REV provirus in the positive samples, two PCRs were developed to amplify fragments of the *gag* + polymerase (*pol*) and envelope (*env*) genes (Table 1), according to the REV sequences of the complete genomes that are available at the GenBank (Table 2). In both cases, a 25 µL PCR was performed, including 50 ng of template DNA, 0.2 mM of each dNTP, 2 mM MgCl_2_, 0.6 µM of each primer, 1× PCR buffer, and 0.75 U of Platinum *Taq* DNA Polymerase (Thermo Fisher Scientific, Carlsbad, CA, USA). The thermal conditions included an initial denaturation step at 94 °C for 3 min, followed by 35 cycles of 94 °C for 45 s, 60 °C for 45 s, and 72 °C for 90 s, followed by a final elongation step at 72 °C for 10 min.

### 2.6. Sequencing and Phylogenetic Analysis

The PCR product of gB, *gag* + *pol*, and *env* from the respective positive control (MDV vaccines) and the sample with the highest concentration were purified with the IllustraTM GFX PCR and Gel Band Purification Kit (GE Healthcare Bio-Sciences, Piscataway, NJ, USA) according to the manufacturer’s instructions. Sequencing was performed on an ABI 3730 DNA Analyzer with the BigDyeTM Terminator v3.1 Cycle Sequencing Kit (Applied Biosystems, Thermo Fisher Scientific, Carlsbad, CA, USA). The sequencing products were assembled using Geneious Prime^®^ 2019.0.4. (www.geneious.com).

The sequences of glycoprotein B from the MDV samples and vaccines were analyzed together with other previously published MDV sequences (Table 3). Partial REV sequences corresponding to the *pol* and *env* genes were analyzed and compared with the reference sequences (Table 2). Multiple sequence alignments were performed using Clustal W [20], and an identity matrix of nucleotides and inferred amino acids was generated using Geneious Prime^®^ 2019.0.4. The selection of the best-fit substitution models and the construction of phylogenetic trees were performed using MEGA v7.0 [21].

The sequence data generated in this study was submitted to the GenBank with the following accession numbers: USP386-MDV (MH825642), CVI988 (MH825643), SB-1 (MH825644), HVT (MH825645), and USP386-REV (*gag* + *pol*: MH673475; *env*: MH673476).

## 3. Results

### 3.1. Histopathologic Examination

**Figure 2:**
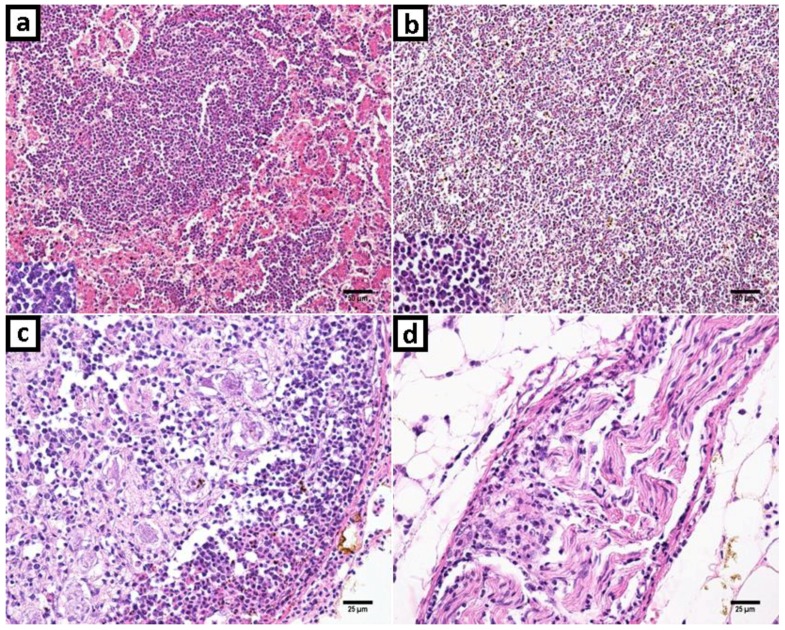
Marek’s disease and reticuloendotheliosis in histopathology with hematoxylin and eosin (H&E) stanning. (**a**) Liver: Proliferation and infiltration of neoplastic small to medium size lymphocytes that replaced liver parenchyma. The neoplastic cells were uniform and small lymphocytes. (**b**) Spleen: Neoplastic lymphoid cells expanding and obliterating histologic structures; and with a highlight in detail of lymphocytes. (**c**) Ganglion: Neoplastic uniform small lymphocytes infiltration and hemorrhage foci. (**d**) Peripheric nerve. Increased cellularity due to small lymphocytes infiltration.

Marked uniform small to medium size neoplastic lymphocytes were infiltrating and replacing multifocal to diffuse the liver and spleen parenchyma (Figure 2a,b). There were multifocal neoplastic lymphoid cells in the kidney and lung. Trachea, proventriculus, gizzard, and small intestine lamina propria showed increased thickness by uniform neoplastic lymphocytes infiltration. Peripheral nerves and ganglion were moderate to marked infiltrated by uniform neoplastic lymphocytes, as showed in Figure 2c,d, respectively. There were few heterophils and plasma cells among neoplastic lymphocytes in all organs examined. Thymic atrophy also was observed in part and there were lymphocytes arranged in focal nodules.

### 3.2. Detection of MDV, REV and ALV through PCR examination

A PCR product of 226 bp corresponding to a fragment of the pp38 gene was detected in all four DNA samples analyzed (liver, spleen, proventriculus, and small intestine) and the positive control. Generic PCR for the gB gene tested positive and amplified a fragment of 728 bp in all the samples and the positive controls for 3 MDV serotypes. According to real-time PCR analysis, Cycle threshold (Ct) values for the non-CVI988 reaction were 19.28 in liver, 18.70 in spleen, 25.58 in proventriculus, 22.72 in small intestine, and undetermined for Rispens vaccine and for negative control. For CVI988-specific reaction, all the organ samples and the negative control did not amplify (Ct values undetermined) while for Rispens vaccine the Ct value was 10.88.

In the case of REV, a PCR product of 201 bp corresponding to a fragment of LTR was detected in all four DNA samples analyzed as well as the positive synthetic control. PCRs designed to amplify the junction between the *gag* + *pol* genes and the last part of the *env* gene tested positive and amplified fragments of 767 and 703 bp, respectively, in all organ samples. All MDV positive controls tested negative in all REV-specific PCRs. The RT-Nested-PCR reactions for ALV were negative for all the organ samples and positive in the synthetic control.

### 3.3. Sequence and Phylogenetic Analysis

**Figure 3:**
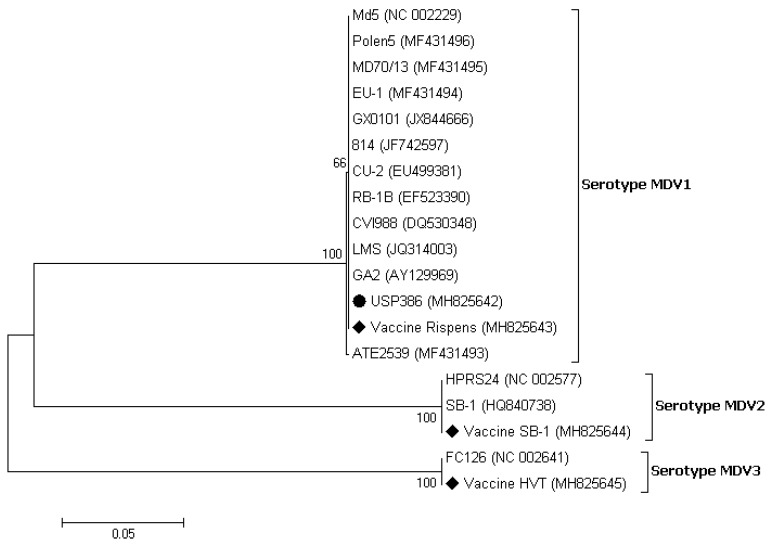
Phylogenetic analysis of the nucleotide sequences of the MDV strains based on the partial gB gene. The strain names and GenBank accession numbers are shown. The black circle represents the field MDV strain used in this study. The black rhombus represents the MDV vaccinal control strains. The phylogenetic tree was constructed in MEGA v7.0 using the Neighbor-Joining method with 1000 bootstrap replicates. The evolutionary distances were computed using the Kimura 2-parameter model (K2 + G + I), and the scale bar represents the number of base substitutions per site.

**Figure 4:**
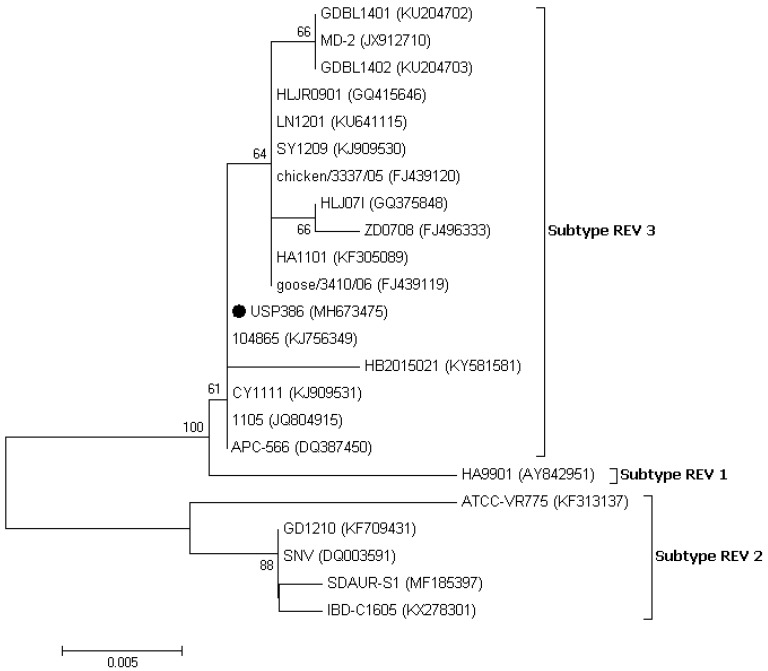
Phylogenetic analysis of the nucleotide sequences of the REV strains based on the partial *pol* gene. The strain names and GenBank accession numbers are shown. The black circle represents the field REV strain used in this study. The phylogenetic tree was constructed in MEGA v7.0 using the Neighbor-Joining method with 1000 bootstrap replicates. The evolutionary distances were computed using the Kimura 2-parameter model (K2), and the scale bar represents the number of base substitutions per site.

**Figure 5:**
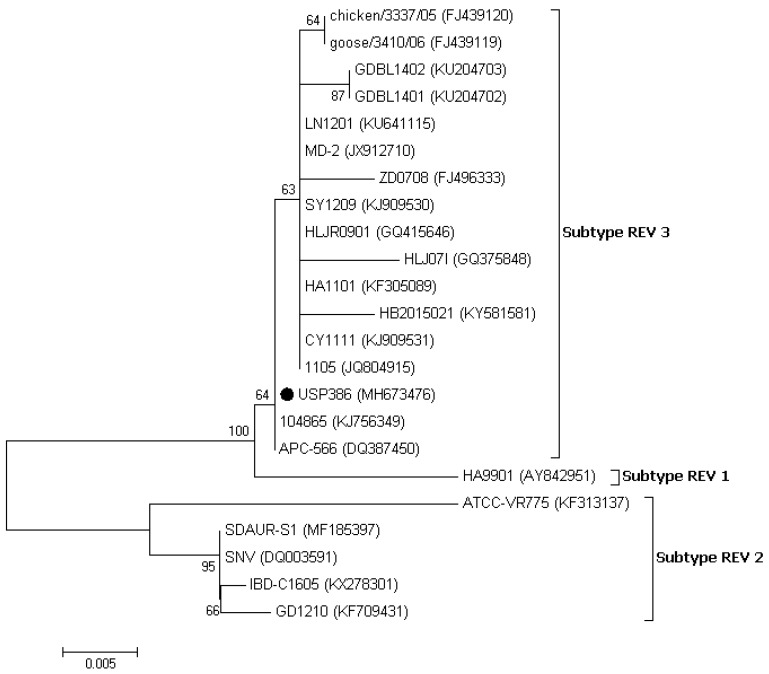
Phylogenetic analysis of the nucleotide sequences of the REV strains based on the partial *env* gene. The strain names and GenBank accession numbers are shown. The black circle represents the field REV strain used in this study. The phylogenetic tree was constructed in MEGA v7.0 using the Neighbor-Joining method with 1000 bootstrap replicates. The evolutionary distances were computed using the Kimura 2-parameter model (K2), and the scale bar represents the number of base substitutions per site.

The similarity analysis between the USP386 MDV sequence with respect to the reference sequences grouped by serotype showed the highest similarity with the MDV1 serotype at the nucleotide level (99.8–100%) and at the amino acids level (100%) (Table 4). The phylogenetic analysis showed that the sequence corresponding to USP386 MDV (MH825642) was found in the cluster corresponding to the MDV1 serotype and the positive control of the same serotype (vaccine Rispens, MH825643). Similarly, the positive controls of serotype 2 (vaccine SB-1, MH825644) and serotype 3 (vaccine HVT, MH825645) were grouped in the corresponding clusters with 100% bootstrap support in all cases (Figure 3). The analysis of similarity in the case of the *pol* gene of REV for the sample USP386 showed greater similarity with the subtype REV3 at the nucleotide level (99.5–100%) as well as at the amino acid level (98.9–100%). Similarly, the *env* gene analysis revealed greater similarity with the REV3 subtype at the nucleotide level (99.2–100%) as well as at the amino acid level (98.5–100%) (Table 5). The phylogenetic analysis for the REV *pol* gene (Figure 4) showed that the grouping of the sequence corresponded to the USP386 sample within the REV3 subtype. Likewise, in the case of the *env* gene (Figure 5), the sample USP386 was grouped within the subtype REV3.

## 4. Discussion

The coinfection of MDV and REV in the field case of lymphoma was described for the first time in Brazil. The histopathological findings correspond primarily to Marek’s disease characterized by neoplastic small to medium size lymphocytes infiltrations in several visceral organs as described previously [1,5]. In addition to these lesions, enlargement of the spleen and liver is a frequent finding in cases of MDV infection with increased mortality and have already been reported in cases of coinfection of MDV with REV [9,13,15] as well as with another avian viruses [22,23,24]. All organ samples tested positive in the PCRs for the pp38 and gB MDV genes and the LTR, *gag*, *pol*, and *env* REV genes, indicating the coinfection in all samples examined. Real-time PCR assays based on the SNP of nucleotide 320 in the pp38 gene [19] were used to differentiate the field strains from the Rispens vaccine (CVI988). In this process, the possibility of infection by the MDV1 serotype vaccine was excluded. To further exclude the presence of the MDV2 and MDV3 serotypes, a generic PCR based on the gB gene was designed, as this gene is one of the most conserved genes among the three serotypes, including the field and vaccine strains [25,26,27,28,29,30,31]. Sequencing demonstrated high levels of identity in the amino acid comparison of the gB gene among the three serotypes, and a lower identity in the comparison of nucleotides. A higher level of discrimination at the nucleotide level among the three serotypes was demonstrated in the phylogenetic tree, with bootstrap supports of 100% for each serotype. Thus, this gB fragment can be used for differential diagnosis and molecular typing. The detection of the REV LTR, *gag* + *pol*, and *env* genes suggests that REV was present with the total proviral genome. REV presents a highly conserved genome [9,32,33,34,35,36]. The USP386 REV strain reflects this fact and shows a high percentage of identity in nucleotides and amino acids for the two genes evaluated. Based on the analysis of both genes, there was greater identity with the strains of the REV3 subtype, intermediate identity with REV1, and lower identity with REV2.

In the phylogenetic analysis of the *pol* gene, the Brazilian REV (USP386) was grouped in subtype 3 (REV3) and was shown to be more closely related to strains 104865 (KJ56349), CY1111 (KJ909531), 1105 (JQ804915), and APC-566 (DQ387450). In the phylogenetic analysis of the *env* gene, USP386 was also grouped in the REV3 subtype and was shown to be more closely related to strains 104865 (KJ56349) and APC-566 (DQ387450). The phylogenetic distribution for both genes is consistent with previous reports [9,37,38]. It has been shown that there is no correlation between time, place, or specific host in the distribution of REV; however, most of the strains reported, including the present study, belong to the REV3 subtype [39,40,41]. The occurrence of cases caused only by REV is infrequent. However, the origin and distribution of these viruses are complex and can be influenced by several factors, including the contamination of vaccines with MDV [42], FPV [43,44], IBDV [34], biological products, or free-living birds [39,45,46]. REV can remain undetected for a long time while it is transmitted horizontally through the direct contact of birds or through certain insects [47], integrated into other field viruses [9,10,11,12,48], or transmitted vertically by eggs [49]. Additionally, REV has no specific host or specific geographic location, having been detected in chickens, ducks, turkeys, geese, pheasants, Japanese quails, peafowl, guinea fowls, and Attwater’s prairie chickens in several countries and continents [3]. Particularly, in the case of South America, two serological reports in Peru [16] and Argentina [17] indicated the presumable presence of REV in this region several years ago. However, it will be difficult to determine the source of REV in Brazil until further studies of regional strains are available. The immunosuppressive capacity of REV can significantly influence poultry farming, leading to economic losses associated with reduced efficiency, morbidity, and increased mortality from coinfections with other pathological agents, which can be associated exclusively with the damages. The genome status of REV (partial or integral) in coinfection with other field viruses can lead to alterations in the severity of lesions [10,13,14] and the inefficiency of vaccines [9,15,50].

As reports of the MDV strain in Brazil are scarce, even more in backyard chicken flocks, this study remembers the importance of the vigilance. On the other hand, the apparition of MDV in backyard flocks is a common event [51] with the presence of low and high-virulence pathotypes [52,53]. The pathogenicity of the MDV strain detected in this study could be comparable with the highest virulent ones, based on the percentage of mortality (>10%) and the lymphoproliferative lesions observed in necropsy (Figure 1a,b) and histopathologic examination (Figure 2a–d). Some possibilities arise here, a virulent enough MDV strain could be responsible for the typical clinic manifestations without the participation of REV; or the last could be helping to a less virulent MDV strain to cause the pathogenic effects. However, conclusive evidences about this should be supported by different approaches as experimental infections and additional genomic studies.

## 5. Conclusions

The knowledge of the infective process and pathologies associated with REV is not fully clarified despite having spent many years since its first reports and studies in some countries with developed poultry industry. In fact, it is possible that this virus is circulating in several other countries, but its monitoring can be neglected. In this context, the South American region includes several countries with notable development of the poultry industry, as is the case of Brazil. Despite the “rumors” about some isolated cases of REV in this continent, this study represents the first description of REV with molecular characterization and in coinfection with MDV causing oncogenic manifestation. However, the main limitation of this study was the number of samples. Further studies involving more cases and samples could reveal or discard the presence of these viruses on field nowadays. We believe that the presence of REV in this region represents a serious threat to many avian species due to its wide spectrum of target hosts and may include not only commercial birds but also ornamental birds, pets, and free-living birds.

Due to the absence of a vaccine or treatment against REV, it is very important to ascertain its origin through monitoring so that measures for eradication and prevention must be taken. In addition, Marek’s disease is associated with large economic losses in poultry and can be supplied with pathogenic viruses from the backyard chicken flocks as the presented strain.

Finally, the importance of this study highlights the REV and MDV detection into this region and provide molecular clues for future research about these viruses.

## Figures and Tables

**Table 1:** Detailed list of the primers used in this study.

Primer Designation	Primers Sequence	Target Gene	Location *	Size Product	Reference
MdCv-F MdCv-R	5′-GTGATGGGAAGGCGATAGAA-3′ 5′-TCCGCATATGTTCCTCCTTC-3′	pp38	127525–127506 ^A^ 127300–127319 ^A^	226 bp	[8]
SNV-LTR-F SNV-LTR-R	5′-AATGGTTGTAAAGGGCAGAT-3’ 5’-CTCCTCTCACTGCCAATCT-3′	LTR (REV)	267-286/8012–8031 ^B^ 466–448/8211–8193 ^B^	201 bp	[8]
Leu3.2F Leu7R	5′-GGAAATGTAGTGTTATRCRATACTCTTATG-3′ 5′-ATCCGCTTCATGCAGGTGCTC-3′	LTR (ALV)	7514–7543 ^C^ 7813–7834 ^C^	321 bp	[18]
Leu11F Leu12R	5′-CGTCGATTGGTGGAAGTAAGGTGG-3′ 5′-TCA GGG AAT CGA CGG TCC GGC C-3′	LTR (ALV)	7594–7617 ^C^ 7785–7806 ^C^	213 bp	[18]
CVI988-F Non-CVI988-F MDV pp38-R	5′-GAGGGAGAGTGGCTGTCAAG-3′ 5′-GAGGGAGAGTGGCTGTCAAA-3′ 5′-TCCGCATATGTTCCTCCTTC-3′	pp38	127487–127468 ^A^ 127487–127468 ^A^ 127300–127319 ^A^	188 bp	[19]
MDVgB-gF1 MDVgB-gR1	5′-CCCATRCCGTTRAACAATTC-3′ 5′-GTYCAATTCGCCATGCTCCA-3′	gB	61554–61573 ^A^ 62281–62262 ^A^	728 bp	This study
REV-Pol1-F5 REV-Pol1-R5	5′-ACTCGCCCAGGAGAGTAGAG-3′ 5′-GAATAGTTTCGCGCAGGCTT-3′	gag + pol	2269–2288 ^B^ 3035–3016 ^B^	767 bp	This study
REV-Env3-F12 REV-Env3-R12	5′-GTGCATACTGGCATCAATCG-3′ 5′-CCACATTCCCCACYGCTCTT-3′	env	7050–7069 ^B^ 7752–7733 ^B^	703 bp	This study

* According to reference genomes for ^A^: MDV (NC_002229), ^B^: REV (NC_006934) and ^C^: ALV (Z46390).

**Table 2:** Reference reticuloendotheliosis virus (REV) strains used for the phylogenetic analysis.

Strain Designation	Isolation Year	Source	Country	GenBank Accession Numbers
SNV	1959	Duck	USA	DQ003591
ATCC-VR775	1972	Duck	USA	KF313137
HA9901	1999	Chicken	China	AY842951
APC-566	2005	Chicken	USA	DQ387450
chicken/3337/05	2005	Chicken	Taiwan	FJ439120
goose/3410/06	2006	Goose	Taiwan	FJ439119
HLJ07I	2007	Chicken	China	GQ375848
ZD0708	2007	Chicken	China	FJ496333
MD-2	2008	HVT Vaccine	China	JX912710
HLJR0901	2009	Chicken	China	GQ415646
1105	2011	Duck	China	JQ804915
HA1101	2011	Chicken	China	KF305089
CY1111	2011	Chicken	China	KJ909531
GD1210	2012	Chicken	China	KF709431
SY1209	2012	Chicken	China	KJ909530
LN1201	2012	Chicken	China	KU641115
104865	2014	Turkey	USA	KJ756349
GDBL1401	2014	Pigeon	China	KU204702
GDBL1402	2014	Pigeon	China	KU204703
HB2015021	2015	Chicken	China	KY581581
IBD-C1605	2016	IBDV vaccine	China	KX278301
SDAUR-S1	2017	Chicken	China	MF185397

**Table 3:** Reference Marek’s disease virus (MDV) strains used for the phylogenetic analysis.

Strain	Year	Source	Pathotype	Country	GenBank No.
Md5	1977	Chicken	Very virulent	USA	NC_002229
Polen5	2010	Chicken	Very virulent plus	Poland	MF431496
MD70/13	1970	Chicken	Virulent	Hungary	MF431495
EU-1	1992	Chicken	Very virulent plus	Italy	MF431494
GX0101	2001	Chicken	Very virulent	China	JX844666
814	1986	Chicken	Mild	China	JF742597
CU-2	1968	Chicken	Mild	USA	EU499381
RB-1B	1981	Chicken	Very virulent	USA	EF523390
CVI988	1969	Chicken	Mild	The Netherlands	DQ530348
ATE2539	2000	Chicken	Very virulent plus	Hungary	MF431493
LMS	2007	Chicken	Very virulent	China	JQ314003
GA (att)	1964	Chicken	Virulent	USA	AY129969
HPRS24	2001	Chicken	vaccine	Japan	NC_002577
SB-1	1978	Chicken	vaccine	USA	HQ840738
FC126	1970	Turkey	vaccine	USA	NC_002641

**Table 4:** The nucleotide and deduced amino acid identities of the USP386 and MDV serotypes.

Serotype	Nucleotide Identity (%)			Aminoacid Identity (%)		
	MDV1	MDV2	MDV3	MDV1	MDV2	MDV3
USP386 (MH825642)	99.8–100.0	77.3	75.5	100.0	91.0	89.6

**Table 5:** The nucleotide and deduced amino acid identities of the USP386 and REV subtypes.

Subtype	Nucleotide Identity (%)			Aminoacid Identity (%)		
	REV 1	REV 2	REV 3	REV 1	REV 2	REV 3
USP386 Pol (MH673475)	98.9	97.3–98.0	99.5–100.0	98.9	98.4–98.9	98.9–100.0
USP386 Env (MH673476)	98.5	95.40–96.9	99.2–100.0	98.1	96.6–97.6	98.5–100.0
